# Locally adapting generic rubrics for the implementation of outcome-based medical education: a mixed-methods approach

**DOI:** 10.1186/s12909-022-03352-4

**Published:** 2022-04-11

**Authors:** Takeshi Kondo, Hiroshi Nishigori, Cees van der Vleuten

**Affiliations:** 1 Center for Postgraduate Clinical Training and Career Development, Nagoya University Hospital, 65, Tsurumai-cho, Showa-ku, Nagoya-city, Aichi Japan; 2 School of Health Professions Education, Department of Educational Development and Research, Maastricht University, Maastricht, The Netherlands

**Keywords:** Local adaptation of rubrics, Outcome-based education, Postgraduate education

## Abstract

**Background:**

Rubrics are frequently used to assess competencies in outcome-based medical education (OBE). The implementation of assessment systems using rubrics is usually realised through years of involvement in projects with various stakeholders. However, for countries or specialities new to OBE, faster and more simplified processes are required. In March 2019, Japan introduced nine competencies and generic rubrics of competencies for medical residents. We explored the local adaptation of these generic rubrics and its consequences for assessors.

**Methods:**

The study followed three steps. First, we locally adapted the generic rubrics. This was followed by conducting mixed-method research to explore the effect of the local adaptation. In step two, we examined the correlations between the scores in the locally adapted assessment sheets for supervising doctors and generic rubrics. In step three, we conducted interviews with supervising doctors. The study was conducted in the General Internal Medicine Department of Nagoya University, Japan. In the first step, doctors in the Medical Education Center and other medical departments, clerks, and residents participated. Supervising doctors in the General Internal Medicine Department participated in the second and third steps.

**Results:**

A locally adapted assessment system was developed and implemented in seven months. The scores of the generic rubrics and the adapted assessment tool completed by the supervising doctors showed good correlations in some items as opposed to others, assessed mainly with other tools. Participant interviews revealed that local adaptation decreased their cognitive load leading to consistent ratings, increased writing of comments, and promoting reflection on instruction.

**Conclusions:**

This adaptation process is a feasible way to begin the implementation of OBE. Local adaptation has advantages over direct use of generic rubrics.

**Supplementary Information:**

The online version contains supplementary material available at 10.1186/s12909-022-03352-4.

## Introduction

A rubric is an assessment tool involving a layout of expectations in a matrix [1]. Rubrics provide timely feedback, prepare learners to use detailed feedback, encourage critical thinking, and facilitate communication with others [1]. As rubrics can be used to objectively assess performance, they are employed to evaluate competencies in outcome-based medical education (OBE) [2, 3], where it is common to develop rubrics for milestones [4–6].

In countries that have a long history of OBE implement milestone rubrics in their complex medical education systems, such as the U.S. and Canada, competencies are defined through their descriptions [4, 7], and speciality-specific rubrics for milestones are defined based on these competencies [8]. Sometimes, generic rubrics for milestones are provided to guide the development of speciality-specific rubrics [9], and multiple assessment tools are mapped to determine the scores for the rubrics [8]. Another way to assess milestones is via entrustable professional activities (EPAs) [6], which assess multiple competencies through authentic clinical practice [6]. EPAs allow physicians to assess competencies in a way that better expresses their views on learners’ performance in clinical practice [6]. Learners are assessed through direct observation, chart reviews, or multisource feedback [10], and these sophisticated assessment systems have been developed over years through the collaboration of multiple stakeholders.

In March 2019, Japan’s Ministry of Health, Labour and Welfare introduced in the Guidelines for Medical Residency, nine competencies and generic rubrics to assess medical residents. These competencies are professionalism (including medical ethics), medical knowledge and problem-solving ability, practical skills and patient care, communication skills, practice of team-based healthcare, management of the quality of care and patient safety, medical practice in society, scientific inquiry, and attitudes for life-long and collaborative learning [11, 12]. The generic rubrics indicate the criteria for scoring the competencies on a scale of one to four, with three to four subscales (see Additional file 1: Appendix 1). In Japan, residents rotate multiple departments during their two-year residencies spending one to three months at each, and the rubrics are applied equally across all institutions and departments. Unlike in the U.S. and Canada, most Japanese speciality training programmes do not use speciality-specific milestones, EPAs connected to milestones, or guidelines mapping assessment tools to required competencies. Since Japan’s adoption of the guidelines, we have been urged to implement the new competency-based system. Many institutions, therefore, began to use the generic rubrics as an assessment tool for supervising doctors in all departments [13].

However, using generic rubrics as an assessment tool in the clinical environment poses potential problems. The items and descriptions are usually abstract and vague to ease applicability in a variety of contexts, but this means that these cannot take into account the local context of each clinical environment. It is difficult to use generic rubrics as an assessment tool [14] as learners struggle to understand where they can improve as per the abstract descriptions provided. The inability to account for local context decreases ecological validity [15], rendering the data acquired through direct use of the generic rubrics practically useless towards making summative decisions. Thus, adaptation and not merely adoption of generic rubrics is the key to conducting meaningful assessments.

Although EPAs, speciality-specific milestones, and guidelines to map assessment tools and timings are already available in some countries as OBE methods, their connection to the context of each country prevents them from being simply imported. For example, the Competence by Design Competence Continuum [16], which is used to define milestones in Canada, does not fit the Japanese medical education system, while the competencies defined in the U.S. and Canada also differ from ours. Considering the requirement to implement OBE, a rapid and simple implementation process is required.

The Association of American Colleges and Universities provides Valid Assessment of Learning in Undergraduate Education (VALUE) rubrics to help assessing essential learning outcomes [17]. These provide a generic evaluation framework to localise the generic rubrics to their context [17] through the modification of sentences and elements within them. The process helps faculty and learners understand the stated criteria, as a result the localised variants reflect actual learning in the context [17]. However, localisation of generic rubrics for application in medical environments differs in some respects from localisation in liberal arts education. Adapting generic rubrics of competencies to the clinical environment requires more than just modifying sentences. The mental model, which clinicians use to assess learners’ performance, sometimes does not fit the fragmented assessment categorised by competencies [18]; so, they assess learners with holistic models including multiple domains of competencies [18]. Comprehensive modifications, including the integration of competencies and the modification of items, are thus required to adapt competencies for each local medical environment. Despite the promise of applying OBE by creating a generic rubric and localising it, there is a lack of research on localising a generic rubric in a medical setting, and on the difference such localisation may offer.

Implementing this innovative process calls for some degree of uncertainty [19], and therefore, the integration of continuous improvement via user feedback. In this case, ‘users’ means supervising doctors, learners, and managers. Among these users, we focused on supervising doctors as they are the primary users of assessment tools and are directly affected by these. Analysis of their experiences may also help implementation in other locations. In this respect, we proposed the following research questions: ‘How can we locally adapt generic rubrics for OBE?’ and ‘What is the effect of such local adaptation on supervising doctors as assessors?’ A rapid process to localise generic rubrics will be useful for countries intent on applying OBE, as well as for specialities that are new to such implementation.

## Method

### Ethical considerations

Data collection began after the study was approved by the Ethical Committee of Nagoya University School of Medicine (approval number 2020–0006). All collected data were anonymised.

### Design

This study followed three steps. In the first step, we locally adapted the generic rubrics and conducted mixed-method research to explore the effect of localisation. An explanatory sequential design was used [20]. In step two, we examined the correlation of scores in the assessment sheets for supervising doctors and the generic rubrics. As the sheets and generic rubrics are qualitatively different, the examination of their correlation was not intended to confirm similarity, but to explore the effects of localisation. In step three, we explored the effects of localisation qualitatively.

### Setting

The study was conducted in the General Internal Medicine (GIM) department of Nagoya University Hospital, Japan. In Japan, two years of residency is mandatory for all graduate students seeking to be medical doctors. The residents rotate through internal medicine, emergencies, surgery, paediatrics, gynaecology, psychiatry, community medicine, and other optional sections. Most rotations last four weeks, although some are longer. The residents are usually assessed by one supervising doctor at the end of each rotation. However, they are now assessed more frequently by multiple doctors in the GIM department of our hospital because the department focuses more on education than others. As multiple assessments are undertaken in the department, the GIM department offers a suitable context within which to observe the effects of localisation.

### Step 1: Local adaptation of generic rubrics

#### Local adaptation project team

Three doctors in the Medical Education Center of our institution, 11 doctors from each department, three clerks, and three representatives from among our hospital residents undertook the local adaptation of the generic rubrics.

#### Process for local adaptation

The local adaptation of generic rubrics was conducted as follows and informed by the Competence by Design (CBD) and Accreditation Council for Graduate Medical Education (ACGME) guidelines, and the localisation process of the VALUE rubrics [8, 17, 21].

1. Reviewing the existing curriculum and assessment tools currently in use.
2. Examining the competencies and descriptions included within the generic rubrics.
3. Mapping the assessment timing and assessor of each competency to the curriculum.
4. Developing assessment tools by integrating the generic rubrics and existing assessment tools.
5. Conducting trials to calibrate the new assessment tools.

### Step 2: Quantitative study

Comparison of scores between the localised assessment sheets and the generic rubrics was conducted to reveal the effects of localisation. Every four weeks the locally adapted sheets and the generic rubrics were distributed among the supervising doctors in the GIM department. The doctors were asked to use both tools to assess the residents twice during their eight-week rotations. The supervising doctors assessed the residents individually, unaware of the scores given by other supervising doctors.

The generic rubrics are scored from Levels 1 to 4 for each competency, with steps of 0.5. Level 1 represents the level of medical school graduation or the starting level for residents. Level 3 represents sufficient competency level to finish residency, and Level 4 represents aspirational achievement (see Table 1; see Additional file 1: Appendix 1 for all rubrics).

**Table 1:** Generic rubric for professionalism (all rubrics are listed in Additional file 1: Appendix 1)

1. Professionalism: Recognise and act appropriately on ethical issues related to medical treatment, research, and education									
Level 1 Medical school graduation		Level 2			Level 3 Expected level at the end of residency			Level 4	
■Is able to outline the historical flow of medicine and medical treatment, clinical ethics, ethical issues related to life and death, and norms related to various ethics		Demonstrates respect for the dignity of the person and the inviolability of life			Protects the dignity of the person and respects the inviolability of life			Acts as a role model to others	
		Gives minimum consideration to patient privacy and confidentiality			Respects patient privacy and maintains confidentiality			Acts as a role model to others	
■Is able to explain the significance and necessity of patients' fundamental rights, the significance of the right to self-determination, patients' values, and informed consent and assent		Recognises the existence of ethical dilemmas			Recognises ethical dilemmas, and responds based on mutual respect			Recognises ethical dilemmas and makes multifaceted decisions and responses based on mutual respect	
		Recognises the existence of conflicts of interest			Recognises conflicts of interest and deals with them according to management policy			Acts as a role model to others	
■Understands the significance and necessity of the right to self-determination, patient values, informed consent, and informed assent, etc. Considers patient privacy, understands the importance of confidentiality, and of handling patients appropriately		Recognises the need to ensure transparency and to prevent misconduct in medical treatment, research, and education			Ensures transparency in medical treatment, research, and education, and strives to prevent misconduct			Acts as a role model to others	
□	□		□	□		□	□		□
□ no chance to observe									
Comment:									

In step 1, we mapped some of the competencies to other assessment tools and created items to assess multiple competencies. Consequently, the locally adapted assessment tools did not contain all competencies and did not parallel the items in the generic rubrics. Following this, we developed conversion formulas to understand the correlation between the generic rubrics and localised tools. The scores were compared using Spearman's correlation.

Data collection was conducted from May 2020 to February 2021. Four supervising doctors in the GIM department completed a total of 20 sets of both the generic rubrics and the localised assessment sheet. A total of 10 out of 16 residents were assessed during the study period.

### Step 3: Qualitative study

Interviews informed by the quantitative results were conducted with the assessors to explore the effects of localisation.

#### Participants

Supervising doctors in the GIM department who used both the generic rubrics and localised tools participated. Multiple supervising doctors assessed the residents every four weeks; however, only doctors who agreed to participate in this study were included.

#### Methodology

The first author conducted interviews with each participant in a quiet room based on an interview guide (Additional file 2: Appendix 2). Four supervising doctors were reqruited as interviewees.

#### Analysis

The interviews were audio-recorded, and the data were transcribed and de-identified after each interview. Thematic analysis was performed on the text data by the first author [22], informed by Braun and Clarke’s six-phase framework [22]. After each interview, the author noted down early impressions, and the transcribed text was divided into chunks. The analysis aimed to specifically answer the study’s research questions; resulting in a theoretical/thematic, rather than inductive analysis [22]. After identifying and coding relevant text chunks, the next interview was conducted. After the completion of the interviews, all text and codes were reviewed to identify themes. The first author discussed the codes and themes with the professor of the Education Center, and the themes were then finalised.

#### Reflexivity

The first author was a core member of the project team that localised the generic rubrics. This author, being a medical doctor in the GIM department, was familiar with all participants.

### Patient and public involvement

Patients or the public were not involved in the design or the conduct of our research. We plan to disseminate this paper publicly by introducing it to members of the Japanese Society for Medical Education.

## Results

### Step 1: Results of local adaptation

The local adaptation project began in July 2019. The existing curriculum was examined with a main focus on the assessment system. All assessment tools were collected and scrutinised. The project team then reviewed the generic rubrics to map the competencies to the curriculum. The assessment tools included case reports, reflection sheets, and assessment sheets from supervising doctors, nurses, other medical professionals, and patients. The mapped assessment tools are described in Table 2.

**Table 2:** Mapped assessment tools

Competencies		Assessment tools
C1	Professionalism	Assessment sheet completed by nurses
		Assessment sheet completed by patients
		Assessment sheet completed by other medical professionals
		Reports about unprofessional behaviour
C2	Medical knowledge and problem-solving ability	Assessment sheet completed by supervising doctors (in university hospital, other institutions)
		Short reports of cases by symptom and major disease
C3	Practical skills and patient care	Assessment sheet completed by supervising doctors (in university hospital, other institutions)
		Check sheet for skills in the emergency department
		Short reports of cases by symptom and major disease
C4	Communication skills	Assessment sheet completed by supervising doctors (in university hospital, other institutions)
		Assessment sheet completed by nurses
		Assessment sheet completed by patients
		Assessment sheet completed by other medical professionals
C5	Practice of team-based healthcare	Assessment sheet completed by Emergency Department nurses
		Assessment sheet completed by nurses
		Assessment sheet completed by patients
		Assessment sheet completed by other medical professionals
C6	Management of quality of care and patient safety	Incident reports
		Reports about unprofessional behaviour
		Assessment sheet completed by Emergency Department nurses
		Assessment sheet completed by supervising doctors (in university hospital, other institutions)
C7	Medical practice in society	Assessment sheet completed by nurses
		Assessment sheet completed in community hospitals and clinics
C8	Scientific inquiry	Assessment sheet completed by supervising doctors (in university hospital, other institutions)
		Reports of attendance of academic conferences
		Short reports of cases by symptom and major disease (discussion part)
C9	Attitudes for life-long and collaborative learning	Assessment sheet completed by supervising doctors (in university hospital, other institutions)
		Reflection sheet for residents
		Notes on discussions with mentor

In then developing the assessment tools, one resident team member suggested that comments from educators would be useful for learning. We, therefore, included many comment sections within the tools.

After the assessment tools were calibrated through trials, new assessment systems were gradually implemented starting in April 2020. Complete implementation was predicted to take three years.

### Step 2: Quantitative results

Four supervising doctors completed a total of 20 sets of both the generic rubrics and the localised assessment sheet. A total of 10 out of 16 residents were assessed during the study period. We calculated inter-rater reliability based on the time when two supervisors evaluated one resident at the same time. The Cohen kappa was -0.25 and 0.69 for generic rubric and localised tools, respectively, indicating that the localised tools provided a more consistent assessment. Subsequently, we examined correlation using the method shown below. As shown in Tables 2 and 3, not all competencies were assessed in the adapted sheet, and only relevant competencies were compared. A conversion formula, which treated all related items as equal, was used to calculate the scores in the locally adapted assessment sheet (Additional file 3: Appendix 3).

**Table 3:** Items in assessment sheets completed by supervising doctors (the sheet is shown in Additional file 3: Appendix 3)

ID	Item	Score	Competencies^a^
L1	Communication with supervisor	0,1, None	C5
L2	A. Recognising importance of patient safety B. Incident report creation	1 for each	C6
L3	Wording and manner with patients	0,1,2	C4
L4	Needs assessment (Bio/psycho/social)	0,1,2	C4
L5	Informed consent	0,1,2,3	C4
L6	Searching medical knowledge and skills new for learner	0,1,2,3	C6
L7	Accepting feedback from others	0,1,2	C9
L8	Reflection	0,1,2,3	C9
L9	Attendance of academic meetings	comment	C8
L10	List of entrustable tasks	comment	C2, C3
L11	Rate the tasks in L10	1,2,3,4,5	C2, C3
L12	Points well done	comment	
L13	Points to improve	comment	
L14	Other comments	comment	

^a^C1 to C9 represent the IDs of the competencies described in Table 2

The Spearman's correlation scores of medical knowledge and problem-solving ability, practical skills and patient care, communication skills, practice of team-based healthcare, management of quality of care and patient safety, and attitudes for life-long and collaborative learning were 0.70, 0.70, 0.51, 0.08, 0.04, and 0.61, respectively (Table 4). The scores of medical knowledge and problem-solving ability, practical skills and patient care, communication skills, and attitudes for life-long and collaborative learning were well correlated, although other scores, assessed mainly through other assessment tools (Table 2), did not show significant correlations. In the generic rubrics, the management of the quality of care and patient safety, medicine in the community, and scientific inquiry contained 5 to 50%, and were marked with the option ‘no chance to observe’. These competencies were items either not assessed or assessed mainly by other tools in the locally adapted system.

**Table 4:** Correlation of generic rubrics and localised sheets

ID	Competencies	Spearman's Correlation	*p* Value
C2	Medical knowledge and problem-solving ability	0.64	0.002
C3	Practical skills and patient care	0.59	0.006
C4	Communication skills	0.54	0.013
C5	Practice of team-based healthcare	0.17	0.471
C6	Management of quality of care and patient safety	0.07	0.762
C9	Attitudes for life-long and collaborative learning	0.54	0.015

The correlation of corresponding items suggested that the two tools measured similar competencies. However, the lack of correlation for some items and the high ratio of ‘no chance to observe’ in some generic rubric items indicated that further investigation was neccesary. Consequently, the differences between these two tools were explored qualitatively.

### Step 3: Qualitative results

All four supervising doctors were interviewed after having used both the generic and localised tools. One of the four (Dr. A) advised during the development of localised tools, but the other three were not involved in the development at all. The interviews were conducted from January to February 2021 and lasted around 30 min each.

#### Learning about competencies

As the generic rubrics explicitly state competencies and their descriptions, the supervising doctors stated they could adequately learn from the national guidelines by undertaking assessments. The descriptions in each level and subcategory helped them analyse each of the residents’ competencies:

C-28: ‘I always think that the evaluation is done from various angles, such as medical aspects, human relations, institutional aspects, legal aspects, and so on’

#### Mismatch between the rubrics and clinical context

However, supervising doctors felt that there was a mismatch between their context and the generic rubrics as there were discrepancies between their expectations for residents and the descriptions provided. They felt that the level in some items was not suitable for the residents and believed that some important aspects of their environment were not taken into account:

D-22: ‘I think they don’t appreciate the toughness of facing a patient’s problem, finding the problem, and solving it’

Abstract descriptions also caused mismatches. As the generic rubrics are designed for all departments of all institutions in Japan, expectations as such are generalised. Supervising doctors struggled to understand these descriptions, and had difficulty filling the gaps between the generic rubrics and their clinical context:

A-22: ‘At first, I wasn’t sure what the words meant or how to apply them’

The generic rubrics also contained items that could not be assessed in the GIM department. Moreover, for some competencies, there were items that could both be observed and not observed, thus confusing the supervising doctors.

#### Invalid assessments

The mismatches between the rubrics and the clinical context caused invalid assessments. The supervising doctors emphasised upon the difficulty to maintain consistency with abstract descriptions, and feared their assessments could be affected by these conditions:

A-64: ‘I think there are some things that are not being evaluated properly because people don’t actually understand the contents and just give a random rating’

Items that could not be feasibly assessed in the department confused the supervising doctors and led to invalid ratings:

C-31: ‘I check the box that says “no opportunity for observation”, or I sometimes give a rating somewhere in the middle as a kind of compromise’

#### Inhibition of reflection

The presence of numerous items, including the abstract ones and those that could not be assessed, caused increased cognitive load on the supervising doctors. They, after completing the items, were too exhausted to write further comments. Hence, they completed the sheet but failed to reflect on their education:

D-52: ‘In this case, I was more concerned with checking the abilities of the residents at the time, but I didn’t think there was much I could do about that’

#### Decreased cognitive load resulting from local adaptation

The locally adapted tools were designed to fit the assessors’ clinical context. Although the supervising doctors were part of the development team, the descriptions in the tools were easy for them to understand and select. This enabled the supervising doctors to assess residents with less cognitive load, and they felt this led to more consistent ratings:

B-48: ‘The localised version is more specific, and the level of the evaluation is not ambiguous’

#### Promotion of reflection on instruction

The low cognitive load combined with the efficacy of sentences in the locally adapted tools promoted comment-writing in the supervising doctors. They stated that they could include what they wanted to convey through such comments and could also reflect on their education in this way. The review, in turn, led to future plans:

D-52: ‘Using the localised version, I was able to review what my residents were able to do and what they were not yet able to do. Through this, I was able to figure out what I need to teach again when I teach the same residents in the next term’

#### Avoidance of differentiation by assessment without context

Some supervisors felt that localised tools could not differentiate among the residents, although quantitative results signified otherwise. This is because similar culture within the development team might have affected the tools’ ability to differentiate. The supervisors also demonstrated avoiding differentiation by means of assessment. They especially feared that assessment scores would be presented without context:

C-66: ‘I do think that if someone checks [the box that indicates] that a resident can’t do something, there is a reason why they can’t do it’

## Discussion

This study revealed the process and effects of a local adaptation of Japan’s generic rubrics for implementing OBE. The process enabled us to use a localised assessment system in just seven months. Comparisons of the scores between the generic rubrics and the localised tools revealed good correlations for some items but no correlations for others. Though the direct use of the generic rubrics taught supervising doctors the requirements stipulated by the national guidelines, the rubrics’ abstract descriptions combined with the inability to feasibly assess some items within the department resulted in significant cognitive load and inconsistent assessments. This further inhibited comment-writing and reflection among the assessors. Localisation, by contrast, decreased the cognitive load of assessors and promoted comment writing as well as assessor reflections.

Albeit some differences exist, the design of this study was broadly informed by ACGME and CBD. In ACGME systems, rubrics for speciality-specific milestones have been developed through lengthy projects, and each programme develops an assessment system for milestones combining multiple assessment tools [8]. In CBD, EPAs based on competencies and milestones are developed for each speciality, and each programme implicates EPAs with multiple assessment tools [21]. Conversely, the local adaptation process in this study directly mapped the generic rubrics to the local context. Considering the lengthy process of developing speciality-specific milestones and EPAs, such localising processes are a feasible way to begin implementing OBE in countries or introducing them in specialities. This process has the advantage of directly employing existing generic rubrics.

The study’s local adaptation of the generic rubrics led to valid and consistent assessment ratings and decreased the supervising doctors’ burden, leading to reflection among them. The finding may be explained in terms of working memory and cognitive load. Working memory can only process a limited amount of information at once; in cognitive load theory, three types of cognitive load impact working memory: intrinsic (essential to the task), extraneous (not essential to the task), and germane (load imposed by the learner’s deliberate use of cognitive strategies to facilitate learning) [23]. Filling the gaps between the abstract sentences of the generic rubrics and the local context, while simultaneously considering items suitable for the local context, cause extraneous cognitive load. This load inhibits the use of the germane load, which comprises schema construction through reflection. The process of localisation fills the gap between generic rubrics and local contexts during the developmental process, thus decreasing extraneous load and promoting reflection among supervising doctors.

The correlation of scores for some competencies suggested that both the localised and generic tools assessed similar competencies. However, the lack of correlation in others suggested assessment with other tools. The qualitative interviews also suggested that some items could not feasibly be assessed, resulting in invalid ratings, and implying that the scores given in the generic rubrics may not be appropriate. The results also revealed the risk of directly using generic rubrics, which is one tool to assess all competencies, thereby leading to invalid assessment. On the other hand, our local adaptation process mapped multiple assessment tools to obtain a holistic view of learners. Integrated assessments using multiple tools are thus required for the implementation of OBE.

However, some points in this process should be considered with caution. Localisation processes are directly affected by the development team; in our case, our tendency to avoid differentiation by assessment may have affected our tools. Such a tendency would be derived from a sense of the collective rather than individual efficacy [24]; thus, reflexivity is important in the process.

### Limitations

We selected only one of the assessment tools we developed. This might have harmed the credibility of our study, limiting the scope of the assessment system. However, as the assessment sheet completed by supervising doctors was the first tool we implemented, exploring the effect of the tool informed ways we could implement other tools. In addition, many Japanese institutions currently supply the generic rubrics directly to their supervising doctors. Therefore, a comparison between the generic rubrics and the assessment sheet used by supervising doctors is useful to depict the differences between our newly proposed methods and the current assessment process in Japan.

In the quantitative part of the study, we could only compare some of the assessed competencies as the assessment tool used by the supervising doctors did not cover all competencies. In addition, 20 datasets is a relatively small number and has an impact on both reliability and validity. Nevertheless, the purpose of the comparison was not to conduct a strict statistical analysis, but rather to suggest the effects of localisation; therefore, our comparison of these two tools still revealed useful insights.

In the qualitative part of the study, the interviewees knew that the interviewer was working on the development of localised tools. This may have affected their reporting of the positive effects of directly using generic rubrics and the negative effects of using the localised tool, as in all interviews the interviewer explicitly asked interviewees to report the advantages of using the generic rubrics and the disadvantages of using the localised tool. The fact that the researcher participated in the localisation process also affected analysis, notwithstanding their detailed knowledge of the tools and context. Further qualitative analysis by an neutral interviewer may withdraw more unfiltered responses in the future.

Although our thematic analysis has the flexibility to adapt a variety of sample sizes [25], the number of study participants was small. Recruiting four supervising doctors who used both tools fit the study’s purpose of exploring the effect of localising tools on their primary users, but involving more participants, including learners, would lead to a richer description of the effects.

## Conclusion

This study developed a process to locally adapt generic rubrics to facilitate the implementation of OBE. This simple process is feasible for countries and specialities intent on implementing OBE. The study, while finding reflexivity to be important in localisation, concluded that local adaptation decreases the cognitive load of assessors and promotes reflection on their instruction.

## Supplementary Information

**Additional file 1.**

**Additional file 2.**

**Additional file 3.**

## Data Availability

The datasets generated and/or analysed during the current study are available in the Dryad repository, https://datadryad.org/stash/share/-Xjawm6uIHMi2uF82FXeYU18hezHXYbOz7_n3byHAoQ

## Abbreviations

OBE: Outcome-based medical education
EPA: Entrustable professional activities
VALUE: Valid Assessment of Learning in Undergraduate Education
GIM: General Internal Medicine
CBD: Competence by Design
ACGME: Accreditation Council for Graduate Medical Education
