# Supplementary material for: Locally adapting generic rubrics for the implementation of outcome-based medical education: a mixed-methods approach
Source: BMC Med Educ. 2022 Apr 11;22:262. doi: 10.1186/s12909-022-03352-4 (PMC8996613; doi:10.1186/s12909-022-03352-4)
Supplement: Supplementary file 1 — Additional file 1. [file 12909_2022_3352_MOESM1_ESM.pdf]

Appendix 1 Generic rubrics

1. Professionalism：
Recognize and act appropriately on ethical issues related to medical treatment, research, and education.

Level1<br>graduation of medical school	Level 2	Level 3<br>Expected level at the end of residency	Level4
■Be able to outline the historical flow of medicine and medical treatment, clinical ethics, ethical issues related to life and death, and norms related to various ethics.<br>■To be able to explain the significance and necessity of patients' fundamental rights, the significance of the right to self-determination, patients' values, informed consent and informed assent.<br>■To understand the significance and necessity of the right to self-determination, patient values, informed consent and informed assent, etc. To consider patient privacy and understand the importance of confidentiality and handle patients appropriately.	Demonstrate respect for the dignity of the human person and the inviolability of life.	Protect the dignity of the human person and respect the inviolability of life.	Demonstrate role models to others.
	Give minimum consideration to patient privacy and maintain confidentiality.	Respect patient privacy and maintain confidentiality.	Demonstrate role models to others.
	Recognize the existence of ethical dilemmas.	Recognize ethical dilemmas and respond based on mutual respect.	Recognize ethical dilemmas and make multifaceted decisions and responses based on mutual respect.
	Recognize the existence of conflicts of interest.	Recognize conflicts of interest and deal with them in accordance with the management policy.	Demonstrate role models to others.
	Recognize the need to ensure transparency and prevent misconduct in medical treatment, research, and education.	Ensure transparency in medical treatment, research, and education, and strive to prevent misconduct.	Demonstrate role models to others.

□	□	□	□	□	□	□

□ no chance to observe

Comment:

2. Medical knowledge and problem-solving ability:
To acquire up-to-date medical and health care knowledge, and to use scientific evidence and experience to solve medical problems.

Level1<br>graduation of medical school	Level 2	Level 3<br>Expected level at the end of residency	Level4
■Identify problems, rank them in terms of importance and necessity, and work with other learners and faculty to find better and more specific ways to solve them. Be able to conduct appropriate self-evaluation and develop	List the basic differential diagnosis and plan the initial response for the common syndromes.	Differential diagnosis and initial response to common syndromes through an appropriate clinical reasoning process.	Provide adequate differential diagnosis and initial response for major syndromes.

strategies for improvement.<br>■Integrate lectures, textbooks, and search information to present their own ideas.	Gather basic information and discuss clinical decisions based on medical findings.	Gather patient information and make clinical decisions that are based on the latest medical knowledge and that take into account the patient's wishes and quality of life.	Gather detailed information about the patient and make clinical decisions that integrate the latest medical knowledge with the patient's wishes and quality of life considerations.
	Develop a medical treatment plan that takes into account all aspects of health, medical care, and welfare.	Develop and implement a medical treatment plan that takes into account all aspects of health, medical care, and welfare.	Formulate a medical treatment plan that takes into account each aspect of health, medical care, and welfare, and implement it taking into account patient background and multidisciplinary cooperation.

□	□	□	□	□	□	□
□ no chance to observe						
Comment:						

3. Practical skills and patient care:
Improve clinical skills and provide medical care that takes into account the patient's pain, anxiety, thoughts and intentions.

Level1<br>graduation of medical school	Level 2	Level 3<br>Expected level at the end of residency	Level4
■To understand the basic clinical skills and perform diagnosis and treatment with appropriate attitude.<br>■Understand basic clinical skills and perform diagnosis and treatment in an appropriate manner.<br>■To be able to prepare medical records in a problem-oriented medical record format and to prepare medical documents as necessary.<br>■To be able to explain about urgent conditions	Securely collect the minimum necessary information on the patient's health status, including psychological and social aspects.	Collect information about the patient's health status, including psychological and social aspects, in an effective and safe manner.	Collect information about the patient's health, including psychological and social aspects, in an effective and safe manner in complex cases.
	Safely implement optimal treatment of basic diseases.	Safely implement the most appropriate treatment for the patient's condition.	Optimal treatment of complex diseases is safely implemented according to the patient's condition.

and chronic diseases.	Appropriate medical records and documentation of medical treatment and its rationale, including at least the minimum necessary information.	Prepare medical records and documents related to medical treatment and its rationale appropriately and without delay.	Able to prepare medical records and documents regarding necessary and sufficient medical treatment and its rationale appropriately and without delay, and to demonstrate role models of description.

□	□	□	□	□	□	□

□ no chance to observe

Comment:

4.Communication skills ;
Establish a good relationship with patients and their families based on the patient's psychological and social background.

Level1<br>graduation of medical school	Level 2	Level 3<br>Expected level at the end of residency	Level4
■To be able to outline the methods, skills and effects of communication.<br>■To be able to establish good human relations and empathize with patients and their families.<br>■To be able to understand and organize psychological and social issues in easy-to-understand terms, giving consideration to the pain of patients and their families.<br>■To be able to explain how to deal with patients' requests.	Treat patients and families with a minimum of wording, attitude, and manner.	Treat patients and families with appropriate wording, politeness, and appearance.	Treat patients and their families with appropriate wording, courtesy, and manner, and in a manner that is appropriate to the situation and the patient's family.
	Organize and explain the minimum necessary information for the patient and family. Support the patient's independent decision-making with the supervising physician.	Organize necessary information for patients and their families, explain it in easy-to-understand terms, and support their independent decision-making.	Organize necessary and sufficient information for patients and their families in an appropriate manner, explain it in easy-to-understand terms, and support patients' independent decision-making after taking medical judgment into account.
	Identify key needs of patients and families.	Identify the needs of patients and their families in terms of physical, psychological, and social aspects.	Identify and integrate the needs of patients and families from physical, psychological, and social aspects.

□	□	□	□	□	□	□

□ no chance to observe

Comment:

5. Practice of team-based health care:
Understand the roles of all people involved with the patient and family, including medical professionals, and work together.

Level1<br>graduation of medical school	Level 2	Level 3<br>Expected level at the end of residency	Level4
■To be able to explain the significance of team medicine and to participate in medical treatment as a member of the team (as a student).<br>■To be able to recognize one's own limitations and seek assistance from other healthcare professionals.<br>■To be able to explain the role of the physician in team medicine.	Understand the purpose of the organization and team that provides healthcare in a simple case.	Understand the purpose of a healthcare organization or team and the role of each member of the team.	In complex cases, practice with an understanding of the purpose of the organization or team providing health care and the purpose of the team, etc.
	In simple cases, share information and collaborate with each member of the team.	Share information and collaborate with each member of the team.	Proactively share information with each member of the team and collaborate with them to practice the best team medicine.

□	□	□	□	□	□	□

□ no chance to observe

Comment:

6. Management of quality of care and patient safety:
Provide quality and safe medical care for patients and consider the safety of medical personnel.

Level1<br>graduation of medical school	Level 2	Level 3<br>Expected level at the end of residency	Level4
■To be able to explain the importance of individual attention and organizational risk management in preventing medical accidents.<br>■To be able to explain the importance of reporting, communication, and consultation in the medical field, and the illegality of falsifying medical documents<br>■To be able to outline the nature of medical safety management systems and the causes and prevention of healthcare-associated infections.	Understand the importance of quality of medicine and patient safety.	Understand the importance of medical quality and patient safety, and strive to evaluate and improve them.	Recognize, evaluate, and recommend improvements in healthcare quality and patient safety on a daily basis.
	Be able to report, communicate, and consult with an appropriate frequency in daily work.	Practice reporting, communication, and consultation as part of daily work.	Practice reporting, communication, and consultation, and respond to reporting, communication, and consultation.
	Understand the necessity of preventing and responding to general medical accidents.	Prevent and respond to medical accidents and other incidents.	Atypical medical accidents and other incidents are analyzed individually for prevention and post-incident response.

	Understand the need for health care for health care workers and their own health care.	Understand health management for medical personnel (including vaccination and handling of needlestick accidents). Understand health management for medical personnel (including vaccination and handling of needlestick accidents) and strive to manage their own health.	Take care of your own health and the health of other medical personnel.

□	□	□	□	□	□	□
□ no chance to observe						
Comment:						

7. Medical practice in society:
Understand various medical systems and systems based on the importance of the social aspect of medicine, and contribute to local and international communities.

Level1<br>graduation of medical school	Level 2	Level 3<br>Expected level at the end of residency	Level4
■To be able to outline the current state of medical care and the uneven distribution of physicians in local communities, including remote islands and remote areas.<br>■To be able to explain the current situation of uneven distribution of physicians in local communities, including remote islands and remote areas. To be able to explain medical planning, regional medical concepts, regional comprehensive care, and regional health.<br>■To be able to explain disaster medicine.<br>■Actively participate in and contribute to	Understand the laws, regulations, and systems related to health care.	Understand the purpose and structure of laws and systems related to health care.	Understand the purpose and structure of laws and systems related to health care and apply them to actual clinical practice.
	Understand the system of health insurance and publicly funded medical care.	Appropriately utilize health insurance and publicly funded medical care, while giving consideration to the patient's burden of medical costs.	Determine whether or not health insurance and publicly funded medical care are applicable and utilize them appropriately.
	Understand the importance of understanding the health issues and needs of the community.	Identify local health problems and needs, and propose necessary measures.	Identify community health issues and needs, and propose and implement necessary measures.
	Understand the necessity of preventive medicine, health care, and health promotion.	Strive for preventive medicine, health care, and health promotion.	Provide concrete suggestions for improvement in preventive medicine, health care, and health promotion.
	Understand the comprehensive community care system.	Understand the comprehensive community care system and contribute to its promotion.	Understand the comprehensive community care system and actively participate in its promotion.
	Understand that extraordinary medical needs, such as disasters and infectious disease pandemics, can occur.	Prepare for extraordinary medical demands, such as disasters and infectious disease pandemics.	Assume extraordinary medical demands, such as disasters and infectious disease pandemics, and respond to them in practice leading an organized response.

community healthcare (as a student).			

□	□	□	□	□	□	□

□ no chance to observe

Comment:

8. Scientific Inquiry:
Understand the scientific approach in medicine and health care, and contribute to the development of medicine and health care through academic activities.

Level1<br>graduation of medical school	Level 2	Level 3<br>Expected level at the end of residency	Level4
■To be able to explain that research is conducted for the development of medicine and medical care and for the promotion of patients' benefit.<br>■To be able to use information and knowledge gained from life science lectures, practical training, and analysis of patients and diseases to deepen their understanding, diagnosis, and treatment of diseases.	Recognize medical questions.	Translate medical questions into research questions.	Translate medical questions into research questions and develop a research plan.
	Understand the scientific research method.	Understand and use the scientific research method.	Utilize and practice scientific research methods in accordance with their objectives.
	Understand the significance of clinical research and clinical trials.	Understand the significance of and cooperate with clinical research and trials.	Understand the significance of clinical research and clinical trials, and cooperate and conduct them in actual clinical practice.

□	□	□	□	□	□	□

□ no chance to observe

Comment:

9. Attitudes for life-long and collaborative learning:
To continue to learn autonomously throughout one's life, reflecting on improving the quality of medical care, studying together with other doctors and medical professionals, and being involved in the training of future generations.

Level1<br>graduation of medical school	Level 2	Level 3<br>Expected level at the end of residency	Level4
■To be able to explain the importance of lifelong learning and collect information necessary for continuous learning.	Recognize the need to absorb rapidly changing and developing medical knowledge and technology.	Strive to absorb rapidly changing and developing medical knowledge and technology.	To constantly self-reflect and strive for self-improvement in order to absorb rapidly changing and developing medical knowledge and technology.

	Maintain an attitude of learning from colleagues, junior colleagues, and non-physician medical professionals.	Teach and learn from each other, colleagues, juniors, and non-physician medical professionals.	Study with colleagues, juniors, and non-physician medical professionals while training their successors.
	Recognize the importance of domestic and international policies and the latest trends in medicine and medical care (including drug-resistant bacteria and genomic medicine).	Understand the latest trends in domestic and international policy, medicine, and medical care (including drug-resistant bacteria and genomic medicine).	To understand the latest trends in domestic and international policies, medicine and medical care (including drug-resistant bacteria, genomic medicine, etc.) and apply them to actual clinical practice.

□	□	□	□	□	□	□
□ no chance to observe						
Comment:						
