# Supplementary material for: Locally adapting generic rubrics for the implementation of outcome-based medical education: a mixed-methods approach
Source: BMC Med Educ. 2022 Apr 11;22:262. doi: 10.1186/s12909-022-03352-4 (PMC8996613; doi:10.1186/s12909-022-03352-4)
Supplement: Supplementary file 2 — Additional file 2. [file 12909_2022_3352_MOESM2_ESM.pdf]

Interview guide for

'What is the difference between local and global rubric'

September 9, 2020, Last modified February 22, 2021
Takeshi Kondo from Japan(i6196203)

Prerequisite

Venue: a small meeting room (non-distracting, quiet)

Equipment needed: Tables, chair, voice recorder, notebook and pen to take notes, refreshments

Participants: supervising doctors in general internal medicine department, Nagoya University, Japan

Number of participants: one for each interview(total four)

Participant Consent: A consent form is provided for participants. One copy should be given to participants to keep for their records. The second copy should be kept by the researcher. Allowed to read the information provided prior to signing the consent form

Demographic data: Simple questionnaires to collect anonymous demographic data from participants.

Introduction

I welcome you all to the interview. Thank you for taking time out from your busy schedule.

The objective of this study is to know what is the difference between local and global rubric. Please note that for accurate collection of data for analysis, this interview will be audio-taped and notes will be taken. The data from this study will be published as an academic article and guide the future revision of our assessment. Please speak clearly and audibly one by one so that the recordings are clear for interpretation. These recorded sessions are used only for the research purpose and kept confidential.

Let me familiarize you with some rules. Please turn off your mobile device or keep them in silent mode. If you wish to withdraw from the interview, please let us know.

Coffee, tea, snacks are available on the table for your use.

Let us know if anyone has any questions.

Starting Questions

1. Can you tell me your role in the general internal medicine department.
2. Please tell me how long you have been in the department?

Introductory Questions

1. Please tell me your general feeling about the assessment.
2. Please tell me the assessment you related in the past.

Key Questions

1. How did you feel about global rubric?
    a. What are the advantages of the global rubric?
    b. What are the weak points of the global rubric?
2. How did you feel about localized rubric?
    a. What are the advantages of the localized rubric?
    b. What are the weak points of the localized rubric?
3. What do you think is the difference between global and localized rubric?
4. What do you expect to be the future assessment tools?

Closing questions

1. Please tell me if we have omitted any part of the interview ?

With this I conclude my interview. Thank you for participation.

Guide for the Moderator

1. Do not approve or reject any response, be neutral.

2. Summarize each topic before you move to the next topic.

3. Use the questions as a guide but not adhere to them rigidly.

4. Write down your impression immediately after the interview.
