# Supplementary material for: Locally adapting generic rubrics for the implementation of outcome-based medical education: a mixed-methods approach
Source: BMC Med Educ. 2022 Apr 11;22:262. doi: 10.1186/s12909-022-03352-4 (PMC8996613; doi:10.1186/s12909-022-03352-4)
Supplement: Supplementary file 3 — Additional file 3. [file 12909_2022_3352_MOESM3_ESM.pdf]

Resident assessment sheet by supervising doctors

The information you provide will be used in feedback sessions with residents conducted every few months by their supervisors to help them improve their skills. The cumulative results of the assessment will be used to determine if any changes are necessary in the treatment of residents (e.g., changes in rotations, completion dates, etc.). Basically, it is not possible to change the treatment of residents based on the assessment of only one supervisor, so please describe what you feel.

* Required

1. What is the name of the target resident? *

____

2. What is the name of your department? *

____

3. What is your name? *

____

Quality and Safety of Medical Care

4. Reporting, communication, and consultation *

Mark only one oval.

◯ Appropriate frequency of reporting, communication, and consultation.
◯ Inability to report, communicate, and consult at an appropriate frequency
◯ There was no opportunity for reporting, communication, and consultation.

5. Prevention of and response to medical accidents, etc. (multiple answers possible)

Check all that apply.

- [ ] Understand the necessity of preventing and responding to medical accidents and other incidents.
- [ ] Appropriate incident reporting was done in necessary situations.

Communication

6. Wording, attitude, and appearances. *

Mark only one oval.

- ( ) Appropriate wording, attitude, and appearance when dealing with patients and their families
- ( ) The minimum level of wording, attitude, and appearance for dealing with patients and their families.
- ( ) Lack of minimum wording, attitude, and appearances when interacting with patients and their families.

7. Identifying needs *

Mark only one oval.

- ( ) To be able to understand the needs of patients and their families in terms of physical, psychological, and social aspects.
- ( ) A partial understanding of the needs of patients and their families.
- ( ) The needs of patients and their families are not being understood at all.

8. Explanation (about medical conditions, examination, and treatment) *

It doesn't have to be an extensive explanation of the condition, but it can be as simple as giving the results of a blood test when they come in, or giving the findings of a physical examination.

Mark only one oval.

- To be able to provide patients and their families with easy-to-understand explanations, and also able to support their decision-making.
- To be Able to provide patients and their families with easy-to-understand explanations.
- To be able to provide minimal explanation and communication to patients and their families.
- Not being able to explain to the patient or family, or not being able to communicate at all due to problems in communication

A lifelong commitment to learning together

9. 医学知識・技術の学習 *

Mark only one oval.

- Learned enough new medical knowledge and skills on their own.
- Learned new medical knowledge and skills on my own, even if only partially.
- Demonstrated interest in learning new medical knowledge and skills
- Shows no interest in learning new medical knowledge and skills.

10. Learning from others *

Mark only one oval.

- Accepted feedback from others (senior doctors, colleagues, junior doctors, non-physician medical professionals) and worked to improve.
- Accepted feedback from others (senior doctors, colleagues, junior doctors, non-physician medical professionals)
- Refusing to accept feedback from others (senior doctors, colleagues, junior doctors, non-physician medical professionals)

11. Reflection *

Mark only one oval.

- To be able to reflect on own actions, think of ways to improve them, and implement them.
- To be able to reflect on my own actions and think of ways to improve them.
- To be able to reflect on my own actions
- Not being able to reflect.

Research

12. If the resident has attended or is planning to attend or present at an academic conference related to your department, please provide the date, the name of the conference, and the title of the presentation.

Entrustable activities

13. What tasks (or chores) have you been able to delegate to your residents during this rotation? Please list the main ones in bullet form. *

14. Is the above achievement satisfactory for your residency rotation? *

Mark only one oval.

	1	2	3	4	5	
Very dissatisfied	⬭	⬭	⬭	⬭	⬭	Outstanding

Points that were done well and points for improvement

15. What did the residents who rotated with you do particularly well? *

____
____
____
____
____

16. Please tell us what you would like to see improved in the future for the residents to grow. *

____
____
____
____
____

17. If you have any other comments, please let me know.

____
____
____
____
____

This content is neither created nor endorsed by Google.

Google Forms
